# CompLement C5 Antibodies for decreasing brain injury after aneurysmal Subarachnoid Haemorrhage (CLASH): study protocol for a randomised controlled phase II clinical trial

**DOI:** 10.1186/s13063-020-04838-6

**Published:** 2020-11-25

**Authors:** Inez Koopman, Gabriel J. E. Rinkel, Mervyn D. I. Vergouwen, on behalf of the CLASH study group, I. Koopman, R. W. P. Tack, H. F. Wunderink, A. H. W. Bruns, I. van der Schaaf, K. A. Gelderman, J. P. Greving, A. J. C. Slooter, A. van der Zwan, M. Bartels, G. J. E. Rinkel, M. D. I. Vergouwen

**Affiliations:** Department of Neurology and Neurosurgery, UMC Utrecht Brain Centre, University Medical Centre Utrecht, Utrecht University, Bolognalaan 2-48, 3584 CJ Utrecht, the Netherlands

**Keywords:** Subarachnoid haemorrhage, Eculizumab, Efficacy, Proof-of-concept, Safety, Complement, Inflammation

## Abstract

**Background:**

The inflammatory response after aneurysmal subarachnoid haemorrhage (aSAH) has been associated with early brain injury, delayed cerebral ischaemia, poor functional outcome, and case fatality. In experimental SAH studies, complement C5 antibodies administered shortly after SAH reduced brain injury with approximately 40%. Complement component C5 may be a new therapeutic target to reduce brain injury and hereby improve the outcome after aSAH. We aim to investigate the pharmacodynamic efficacy and safety of eculizumab (complement C5 antibody) in patients with aSAH.

**Methods:**

A randomised, controlled, open-label, phase II clinical trial with blinded outcome assessment. Eculizumab (1200 mg) is administered intravenously < 12 h, on day 3 and on day 7 after ictus. Patients in the intervention group receive prophylactic antibiotics for 4 weeks, and those with a central line or an external ventricular shunt and a positive fungal or yeast culture also receive prophylactic antifungal therapy for 4 weeks. The primary outcome is C5a concentration in the cerebrospinal fluid (CSF) on day 3 after ictus. Secondary outcomes include the occurrence of adverse events, inflammatory parameters in the blood and CSF, cerebral infarction on magnetic resonance imaging, and clinical and cognitive outcomes. We aim to evaluate 26 patients with CSF assessments, 13 in the intervention group and 13 in the comparator group. To compensate for early case fatality and inability to obtain CSF, we will include 20 patients per group.

**Discussion:**

The CLASH trial is the first trial to investigate the pharmacodynamic efficacy and safety of eculizumab in the early phase after aSAH.

**Trial registration:**

Netherlands Trial Register NTR6752. Registered on 27 October 2017

European Clinical Trials Database (EudraCT) 2017-004307-51

## Administrative information

Note: the numbers in curly brackets in this protocol refer to the SPIRIT checklist item numbers. The order of the items has been modified to group similar items (see http://www.equator-network.org/reporting-guidelines/spirit-2013-statement-defining-standard-protocol-items-for-clinical-trials/).

**Table:** 

Title {1}	CompLement C5 Antibodies for decreasing brain injury after aneurysmal Subarachnoid Haemorrhage (CLASH): study protocol for a randomised controlled phase II clinical trial
Trial registration {2a and 2b}.	Netherlands Trial Register: NTR6752, https://www.trialregister.nl/trial/6579. Registered on 27 October 2017. European Clinical Trials Database: EudraCT 2017-004307-51 See Additional File 1 for all items in the WHO trial registration data set for this study.
Protocol version {3}	08-07-2020, version 11.0
Funding {4}	Netherlands Organization for Health Research and Development, the Dutch Brain Foundation, and Alexion Pharmaceuticals.
Author details {5a}	Department of Neurology and Neurosurgery, UMC Utrecht Brain Centre, Matthias van Geuns building, room 02.15, University Medical Centre Utrecht, Utrecht University, Bolognalaan 2-48, 3584 CJ Utrecht, the Netherlands Tel: +31-88-7571441. Email: i.koopman-2@umcutrecht.nl
Name and contact information for the trial sponsor {5b}	University Medical Centre Utrecht Heidelberglaan 100 3584 CX Utrecht Tel: +3188 755 5555
Role of sponsor {5c}	Monitoring and management.

## Introduction

### Background and rationale {6a}

Early brain injury and delayed cerebral ischaemia are important determinants of poor outcome after aneurysmal subarachnoid haemorrhage (aSAH) [1]. No treatment exists to reduce early brain injury, and the effects of current strategies to prevent delayed cerebral ischaemia are only modest [2]. The inflammatory response is considered to play a key role in the pathogenesis of early brain injury and delayed cerebral ischaemia after aSAH [3–6]. Previous studies found that the complement cascade is activated in patients with SAH and associated with poor functional outcome [7, 8]. Complement components C3a and C5a are proinflammatory anaphylatoxins that can induce vasoconstriction and activate coagulation [9–11], processes that have been implicated in the pathophysiology of early brain injury and delayed cerebral ischaemia [1, 12]. C5-specific antibodies, which prevent the formation of C5a, have been shown to reduce microglia activation and cell death by 40% in an SAH mouse model [13]. C5 antibodies (eculizumab) are already used for other inflammatory diseases such as neuromyelitis optica and myasthenia gravis [14, 15]. The aim of this trial is to investigate the pharmacodynamic efficacy (proof-of-concept) and safety of eculizumab in patients with aSAH. As this is the first trial to investigate the use of eculizumab in aSAH patients, the effective dosing regimen for aSAH patients is unknown. In our previous study, we found that the C5a concentration in the cerebrospinal fluid (CSF) of aSAH patients is highly increased (> 1400 times) compared to the C5a concentration in CSF from patients with unruptured intracranial aneurysms [13]. We therefore decided upon administration of a high dose of eculizumab (1200 mg) with repeated drug administration to prevent a wash-out effect.

### Objectives {7}

The aim of this trial is to investigate the pharmacodynamic efficacy (proof-of-concept) and safety of eculizumab in patients with aSAH.

### Trial design {8}

The CLASH trial is a randomised, controlled, open-label, phase II clinical trial with blinded outcome assessment (PROBE) to assess the pharmacodynamic efficacy and safety of eculizumab in patients with aSAH.

## Methods: participants, interventions, and outcomes

### Study setting {9}

This study will be conducted at the University Medical Centre Utrecht (UMC Utrecht), a tertiary referral centre.

### Eligibility criteria {10}

The inclusion and exclusion criteria are presented in Table 1.

**Table 1:** Inclusion and exclusion criteria

**Inclusion criteria**	
• SAH confirmed by CT and aneurysm by CTA or DSA	
• Admission to the UMC Utrecht < 12 h after ictus	
• Age 18 years and older	
**Exclusion criteria**	
• Life expectancy < 10 days	
• Pregnant or breastfeeding women	
• Participation in another clinical therapeutic study	
• History of splenectomy or asplenia	
• Haematologic malignancy	
• Patients receiving chemotherapy	
• Patients who will undergo or underwent an organ transplantation	
• Patients with myasthenia gravis, glucose-6-phosphate dehydrogenase (G6PD) deficiency, or tuberculosis	
• Patients who are or will be treated by plasmapheresis or haemodialysis	
• Patient with a creatinine clearance of < 30 or serum creatinine levels of > 169 μmol/l	
• Patients with a known hereditary complement deficiency	
• Patients allergic to eculizumab, proteins derived from mouse products, or other monoclonal antibodies	
• Patients allergic to (prophylactic) antibiotic treatment for *Neisseria meningitidis* (quinolones or ceftriaxone)	
• If on admission, it is likely that the aneurysm can only be treated with extracranial-intracranial bypass surgery	
• If based on head imaging, it will be unlikely that CSF can be obtained at day 3 after ictus	
• Patients with an ongoing infection on admission which is not appropriately treated	
• Patients who were treated > 4 times with antibiotics during the last year	
• Patients on immunosuppressive therapy	

*SAH* subarachnoid haemorrhage, *CT* computed tomography, *CTA* computed tomography angiography, *DSA* digital subtraction angiography, *UMC Utrecht* University Medical Centre Utrecht, *CSF* cerebrospinal fluid

### Who will take informed consent? {26a}

The physician on call will contact the investigators if an eligible patient is admitted. Written informed consent to participate in the study will be asked from the patient or legally authorized representative after the diagnosis is discussed with the patient and/or the legally authorized representative. Consent is asked by the physician on call or the investigators. If the physician on call is too busy, one of the investigators will come to the hospital to ask for consent. The consent form includes information about the study’s rationale, study procedures, and possible benefits and risks. If it will not be possible to obtain consent and administer eculizumab < 12 h after ictus, the patient and/or legally authorized representative will not be approached for trial participation. The patient or representative will receive as much time as needed to decide about trial participation. However, it is noted during the conversation with the patient and/or legally authorized representative that trial participation can only take place if inclusion < 12 h after ictus is possible.

### Additional consent provisions for collection and use of participant data and biological specimens {26b}

Informed consent is asked for the collection and use of participant data and biological specimens. No ancillary studies will be conducted.

## Interventions

### Explanation for the choice of comparators {6b}

The comparator group in our study is composed of patients with SAH who receive care as usual. No placebo treatment and no standard antibiotics or antifungal therapy is administered to the comparator group unless clinically indicated. Although we would have preferred to make use of a placebo comparator for eculizumab, it was not possible to manufacture placebo eculizumab, ciprofloxacin, and fluconazole infusions in our hospital within a reasonable time window and reasonable costs. In addition, because this trial is a phase II proof-of-concept trial with a primary outcome based on laboratory parameters, the absence of a placebo treatment in the comparator group will not affect our primary outcome.

### Intervention description {11a}

**Fig. 1:**
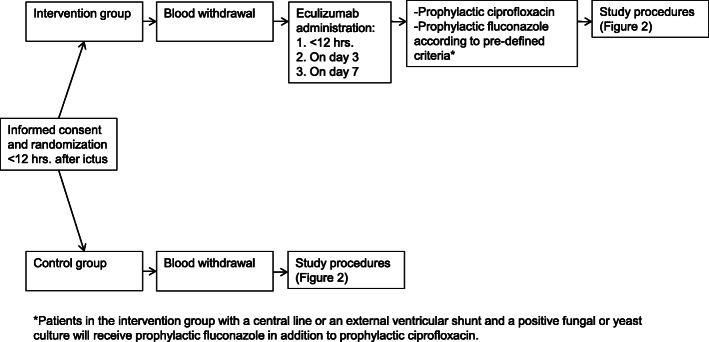
Treatment allocation. hrs. = hours

The intervention consists of intravenous infusion with eculizumab 1200 mg at three different time points: < 12 h, on day 3, and day 7 after ictus (Fig. 1). The day of ictus is defined as day 1. To decrease the risk of (meningococcal) infection due to eculizumab treatment, patients in the intervention group receive prophylactic treatment with ciprofloxacin during the first 4 weeks after ictus. During the recruitment phase, after the inclusion of the 6th patient, we changed our protocol based on a serious adverse event (SAE) that occurred (cerebral fungal infection in a patient with external ventricular shunt). After the amendment, patients in the intervention group with a central line or an external ventricular shunt and a positive fungal or yeast culture receive prophylactic ciprofloxacin and fluconazole for the first 4 weeks after ictus. Throat and rectal swabs are performed weekly in the intervention group during the in-hospital stay to test for fungus or yeast carriership/colonization and (multi-) drug resistance. In consultation with the microbiologist and infectious disease specialist, prophylactic treatment will be switched if (1) swabs are positive for microorganisms that require treatment and are not covered by our prophylactic regimen and (2) resistant microbial phenotypes are found.

### Criteria for discontinuing or modifying allocated interventions {11b}

Treatment with eculizumab will be discontinued in the following events:

- Anaphylactic shock after infusion of eculizumab. In case of a mild allergic reaction, the infusion rate will be slowed down.
- Patients with *Neisseria meningitidis* meningitis, CSF culture-proven.
- Patients with *Neisseria meningitidis* sepsis, blood culture-proven.
- Other medical reasons for which the treating physician or investigator deems it necessary to discontinue treatment.

No modifications to the dosing schedule will be performed except when a mild allergic reaction occurs. If treatment with eculizumab is halted, other study procedures will continue according to the study protocol.

### Strategies to improve adherence to interventions {11c}

The investigator visits the trial participants regularly during their in-hospital stay to explain all study procedures, provided the clinical condition allows such an explanation. If the trial participant is discharged, the participant receives an explanation about the importance of adherence to the study protocol and a safety card to inform other physicians about trial participation. After discharge, trial participants are seen at the outpatient clinic by the same investigator, who then again explains all study procedures.

### Relevant concomitant care permitted or prohibited during the trial {11d}

All concomitant care or interventions are allowed except for the concomitant care listed in the exclusion criteria (e.g. immunosuppressive medication, chemotherapy).

### Provisions for post-trial care {30}

Trial participants will be insured by the UMC Utrecht clinical trial participant insurance.

### Outcomes {12}

**Fig. 2:**
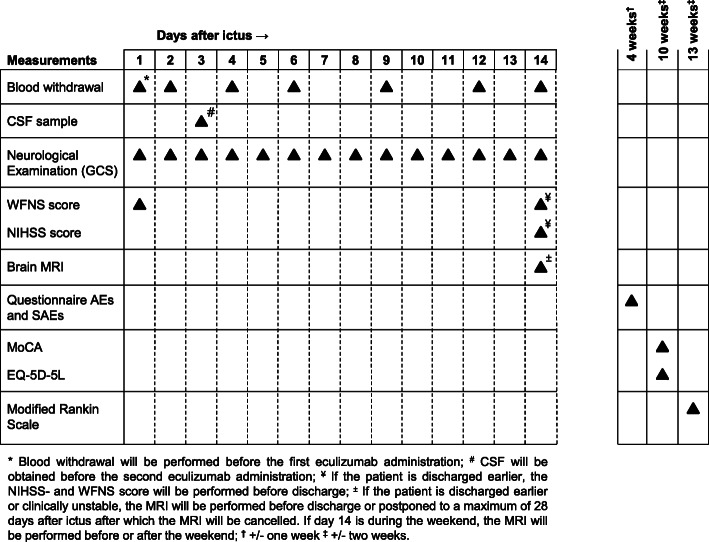
Study procedures. CSF, cerebrospinal fluid; GCS, Glasgow Coma Score; WFNS, World Federation of Neurosurgical Societies; NIHSS, National Institutes of Health Stroke Scale; MRI, magnetic resonance imaging; AEs, adverse events; SAEs, serious adverse events; MoCA, Montreal Cognitive Assessment; EQ-5D-5L, standardized instrument for use as a measure of health outcome

The primary outcome is C5a concentration in CSF on day 3 after ictus. The difference in C5a concentrations between the intervention and comparator groups will be assessed (either the difference in the mean or median concentrations, depending on the distribution of the data). CSF is obtained by either lumbar puncture or sampling from an external lumbar or ventricular drain. Secondary outcomes are listed in Table 2. The mean or median scores between the intervention and comparator groups will be assessed at various time points (Fig. 2) for all secondary outcomes except for the World Federation of Neurosurgical Societies (WFNS) score and Modified Ranking Scale (mRS) score for which the proportion of patients with a specific score will be compared.

**Table 2:** Secondary outcomes

1. The occurrence of AEs and SAEs. Blinded assessment of infections will be performed by an expert panel consisting of a microbiologist and an infectious disease specialist.	
2. Blood and CSF parameters of inflammation.	
3. Eculizumab concentration in the blood and CSF.	
4. Daily neurological condition measured by the GCS.	
5. Neurological condition measured by the NIHSS and WFNS scores on day 14 after ictus. If the patient is discharged earlier, the NIHSS and WFNS scores will be performed before discharge.	
6. Cerebral infarction defined as infarction identified on brain MRI after the exclusion of procedure-related infarctions [16].	
7. Cognition measured by the MoCA.	
8. Quality of life measured by the EQ-5D-5L questionnaire.	
9. Functional outcome measured by the mRS score. Telephone interviews will be conducted by a qualified person who is blinded for allocation.	

*AEs* adverse events, *SAEs* serious adverse events, *CSF* cerebrospinal fluid, *GCS* Glasgow Coma Score, *NIHSS* National Institutes of Health Stroke Scale, *WFNS* World Federation of Neurosurgical Societies, *MRI* magnetic resonance imaging, *MoCA* Montreal Cognitive Assessment, *mRS* Modified Ranking Scale

### Participant timeline {13}

The time schedule of enrolment, intervention, assessments, and visits for participants can be found in Fig. 2.

### Sample size {14}

The sample size calculation is based on a previous study with eculizumab in patients with neuromyelitis optica [14]. In that study, C5 concentration in CSF was measured in 11 patients before and after treatment with eculizumab. In six patients, C5 was undetectable after treatment was started, and in the remaining five patients, the mean C5 concentration in CSF decreased with 58%. For the CLASH trial, we conservatively assumed an overall reduction of C5 concentration in CSF with 55% and extrapolated this to a similar reduction in C5a concentration in CSF. Based on *α* = 0.05 and *β* = 0.20 and a standard deviation of 50%, 13 patients are needed in each group. The group size will be increased to 20 patients per group, taking into account an assumed mortality rate of 25% and 2 patients per group who refuse a lumbar puncture in a later phase despite giving informed consent earlier.

### Recruitment {15}

Eligible study participants are identified by the physician on call. Because consent for trial participation is asked after the diagnosis is discussed, the information about the diagnosis and the trial can be overwhelming for the patient and family. We developed a lay-men 3D medical animation explaining what a subarachnoid haemorrhage is. The medical animation is shown to the patient and/or family on an iPad and facilitates a conversation about trial participation. In addition, the Subarachnoid Haemorrhage Patient Association endorsed our clinical trial, and information about this trial was posted on their website.

## Assignment of interventions: allocation

### Sequence generation {16a}

A computer-generated block randomization is used to randomise patients. No stratification factors were used.

### Concealment mechanism {16b}

Not applicable, this trial is an open-label trial with blinded outcome assessment.

### Implementation {16c}

Patients are randomised by the investigators via a centralized secured website after informed consent from the patient or legally authorized representative is obtained. Assignment occurs computer-generated. Directly after randomization, the investigators receive an email with the allocation. Patients are allocated 1:1 to either (1) the intervention arm or (2) care as usual.

## Assignment of interventions: blinding

### Who will be blinded {17a}

The use of coded samples will allow blinded measurement of C5a concentration by the laboratory technician. In addition, functional outcome measured by the mRS score will be conducted by a qualified person who is blinded for allocation. Blinded assessment of infections will be performed by an expert panel consisting of a microbiologist and an infectious disease specialist. Data analysts will not be blinded to allocation, because this trial is a proof-of-concept trial with a primary outcome based on laboratory parameters.

### Procedure for unblinding if needed {17b}

Not applicable.

### Plans for assessment and collection of outcomes {18a}

Data collection is the same for the intervention and comparator groups (Fig. 2). Each patient is assigned a code upon randomization by a computer. Data are collected from the electronic patient system and then transferred to an electronic case report form (E-CRF) by the investigator under the code assigned upon randomization. If data are not available in the electronic patient system, a paper form is used after which the data are transferred to the E-CRF, also under the code assigned upon randomization. The electronic forms are a mix of pre-existing forms (e.g. GCS, NIHSS, mRS, and EQ-5D-5L) and forms designed by the investigator. Data quality checks are performed by a contract research organization according to the approved monitor plan (25% of all data entered will be checked). In addition, the E-CRF makes use of reference values where applicable. All data will be stored on a secured server.

### Plans to promote participant retention and complete follow-up {18b}

As specified in 11c, the participant receives a face-to-face explanation about the importance of adherence to the protocol. In addition, participants will be asked if their phone number is valid. Study visits to the outpatient clinic are planned on the same day as the patient’s standard of care appointment at the rehabilitation centre. Participants who choose to leave the trial are asked whether the collected data can be used for this research. If permission is provided, their data will be handled as data from other trial participants. If permission is not provided, their data will be deleted from the E-CRF and the paper forms will be destroyed.

### Data management {19}

The decryption key will be stored securely and is only accessible by the investigators. Data management procedures can be found in the data management plan provided as Additional File 2.

### Confidentiality {27}

Data will be pseudonymized to guarantee confidentiality. Pseudonymized patient data will only be accessible by the investigators, the study monitor, and the health authorities, if required. The personal data collected and stored for the purpose of this study will be treated in accordance with the provisions of the General Data Protection Regulation (GPDR: Regulation EU 2016/679). Samples are de-identified and stored under UNI EN ISO 9001: 2015 regulations.

### Plans for collection, laboratory evaluation, and storage of biological specimens for genetic or molecular analysis in this trial/future use {33}

The CSF and blood sampling, processing, storage, and immunoassays are described in Additional File 3.

## Statistical methods

### Statistical methods for primary and secondary outcomes {20a}

The primary analysis will be based on the per-protocol principle in which patients with CSF assessments will be included. Patients in the intervention group with CSF assessments are included if they received the first eculizumab infusion.

#### Primary analysis

Groups will be compared with an independent *t* test or Mann-Whitney *U* test, dependent on the distribution of data. If the proportion of patients categorized according to the Prognosis on Admission of Aneurysmal Subarachnoid Haemorrhage (PAASH) scale and the median Hijdra score differs between the intervention and comparator groups, a multivariable logistic regression analysis with adjustment for these variables will be performed.

#### Secondary analyses

Inflammatory parameters in the blood and daily Glasgow Coma Score (GSC) will be analysed with a linear mixed model. CSF inflammatory parameters in both groups will be compared by means of an independent *t* test or Mann-Whitney *U* test, dependent on the distribution of the data. To compare the NIHSS score, cognition, and quality of life, a chi-square or Fisher’s exact test will be applied. A proportional odds model will be used to assess the effect of eculizumab on WFNS score and mRS score [17].

### Interim analyses {21b}

An interim analysis will be performed based on SAE reporting, outcome, or case fatality after the inclusion of 20 patients. The data safety monitoring board (DSMB) can advise early termination of the trial if there is evidence of severe harm based on SAE reporting, outcome, or case fatality.

### Methods for additional analyses (e.g. subgroup analyses) {20b}

Not applicable.

### Methods in analysis to handle protocol non-adherence and any statistical methods to handle missing data {20c}

Missing data will not be imputed.

### Plans to give access to the full protocol, participant-level data, and statistical code {31c}

The dataset of this study is not publicly available but is available from the corresponding author upon reasonable request.

## Oversight and monitoring

### Composition of the coordinating centre and trial steering committee {5d}

This is a monocentre trial. The trial steering committee consists of multiple experts in the field of subarachnoid haemorrhage and infectious diseases and experts who have experience with treatment of patients with eculizumab. The trial steering committee provides guidance for general trial conduct.

### Composition of the data monitoring committee, its role, and reporting structure {21a}

An independent data safety monitoring board (DSMB) will oversee the safety and overall conduct of the CLASH trial. The DSMB consists of two neurologists and one clinical epidemiologist. The chair has previous experience in serving on DSMBs and experience in chairing meetings.

### Adverse event reporting and harms {22}

Safety will be examined by ongoing monitoring of SAEs and suspected unexpected serious adverse reactions (SUSARs) and by an interim analysis. Listings of infections will be reported every 2 months to the DSMB chairman. The DSMB can advise early termination of the trial if there is evidence of severe harm based on SAE reporting, outcome, or case fatality. SAEs that can be expected based on the patient population include rebleeding, per-procedural aneurysm rupture, hydrocephalus, hyponatraemia, delayed cerebral ischaemia, nosocomial infections, Tersons’s syndrome, epilepsy, and delirium. All AEs, SAEs, and SUSARs will be collected systematically by reviewing the electronic patient system and by reporting of the patient spontaneously or at 4 weeks after ictus. All adverse events will be reported in the supplementary material of the trial’s publication.

### Frequency and plans for auditing trial conduct {23}

A contract research organization will audit trial conduct following the approved monitor plan. This includes 2–3 visits a year in which the in- and exclusion criteria, informed consent forms, source data, and SAEs reporting forms are verified.

### Plans for communicating important protocol amendments to relevant parties (e.g. trial participants, ethical committees) {25}

The coordinating investigator will submit amendments for review to the ethics committee of the UMC Utrecht. Upon approval, all parties involved will be informed about the amendment, and the protocol in the clinical trial registry will be updated.

### Dissemination plans {31a}

After the clinical trial is completed, an article will be written for publication in an international journal. The results will also be presented at an international conference.

## Discussion

In aSAH patients, an inflammatory response occurs in the subarachnoid space shortly after the bleeding. This inflammatory response has been associated with early brain injury, delayed cerebral ischaemia, poor functional outcome, and case fatality [1, 3–5]. In experimental SAH studies, treatment with complement C5 antibodies shortly after SAH reduced brain injury with approximately 40% [13]. Complement component C5 may be an important target to reduce brain injury and hereby improve the outcome after aSAH. The CLASH trial is the first phase II trial to investigate the pharmacodynamic efficacy and safety of eculizumab in aSAH patients.

We chose an open-label design with blinded outcome assessment for several reasons: (1) we deemed it unethical to subject patients in the comparator group to 4 weeks of prophylactic treatment with ciprofloxacin and fluconazole, (2) it was not possible to manufacture placebo ciprofloxacin and fluconazole infusions at our hospital within a reasonable time window and reasonable costs, and (3) this trial is a proof-of-concept trial with a primary outcome based on laboratory parameters. We do not expect that ciprofloxacin or fluconazole will influence the C5a concentration in the CSF. However, the outcomes that are self-reported and assessed by clinicians not blinded to allocation can be biassed by the open-label design.

The CLASH trial is designed as a proof-of-concept trial and is not powered to assess the effectiveness of treatment with eculizumab. Safety is an important outcome of this trial. Eculizumab treatment increases the risk of infection. Central lines or external drains can provide a point of entry for microbes. aSAH patients will therefore be closely monitored during the in-hospital stay and receive prophylactic antibiotics, antifungal therapy if necessary, and a patient safety card with instructions. If our trial demonstrates the efficacy and safety of eculizumab in aSAH patients, the next step will be to plan a phase III trial.

### Trial status

8 July 2019—version 11.0

The first patient has been recruited in October 2018. In September 2020, 20 patients have been enrolled. The recruitment is anticipated to be completed by 1 April 2021, with a follow-up period until 1 July 2021. Protocol modifications will be communicated to relevant parties. The results of this trial will be published in a peer-reviewed scientific journal.

## Supplementary Information

**Additional file 1.** WHO trial registration data set.

**Additional file 2.** Data management plan.

**Additional file 3.** Sampling, processing, storage, and immunoassays.

## Data Availability

The datasets used and/or analysed during the current study are available from the corresponding author upon reasonable request.

## Abbreviations

aSAH: Aneurysmal subarachnoid haemorrhage
CLASH: CompLement C5 Antibodies for decreasing brain injury after aneurysmal Subarachnoid Haemorrhage
CSF: Cerebrospinal fluid
DSMB: Data safety monitoring board
E-CRF: Electronic case report form
GCS: Glasgow Coma Score
mRS: Modified Ranking Scale
PAASH: Prognosis on Admission of Aneurysmal Subarachnoid Haemorrhage
PROBE: Prospective Randomised Open Blinded End-point
SAE: Serious adverse event
SUSAR: Suspected unexpected serious adverse reaction
UMC Utrecht: University Medical Centre Utrecht
WFNS: World Federation of Neurosurgical Societies
ZonMw: Netherlands Organization for Health Research and Development
