# Supplementary material for: CompLement C5 Antibodies for decreasing brain injury after aneurysmal Subarachnoid Haemorrhage (CLASH): study protocol for a randomised controlled phase II clinical trial
Source: Trials. 2020 Nov 25;21:969. doi: 10.1186/s13063-020-04838-6 (PMC7687754; doi:10.1186/s13063-020-04838-6)
Supplement: Supplementary file 1 — Additional file 1. WHO trial registration data set. [file 13063_2020_4838_MOESM1_ESM.docx]

**Additional File 1**

WHO trial registration data set

| **Data category** | **Information**[**^32^**](https://www.spirit-statement.org/spirit-statement/references#32) |
| --- | --- |
| Primary registry and trial identifying number | Netherlands Trial Register: NTR6752. <https://www.trialregister.nl/trial/6579> |
| Date of registration in primary registry | 27 October 2017 |
| Secondary identifying numbers | European Clinical Trials Database: EudraCT 2017-004307-51. |
| Source(s) of monetary or material support | Netherlands Organization for Health Research and Development, the Dutch Brain Foundation, and Alexion Pharmaceuticals. |
| Primary sponsor | University Medical Centre Utrecht |
| Secondary sponsor(s) | Not applicable |
| Contact for public queries | I. Koopman, MD  Tel: +31-88-7571441. Email: i.koopman-2@umcutrecht.nl |
| Contact for scientific queries | I. Koopman, MD  Tel: +31-88-7571441. Email: i.koopman-2@umcutrecht.nl |
| Public title | Inflammation inhibitors to reduce brain injury after a hemorrhagic stroke. |
| Scientific title | CompLement C5 Antibodies for decreasing brain injury after aneurysmal Subarachnoid Haemorrhage (CLASH): study protocol for a randomized controlled phase II clinical trial |
| Countries of recruitment | The Netherlands |
| Health condition(s) or problem(s) studied | Aneurysmal subarachnoid haemorrhage |
| Intervention(s) | Intervention: Eculizumab 1200 mg on day 1,3, and 7 Control: No treatment (care as usual) |
| Key inclusion and exclusion criteria | Ages eligible for study: ≥18 years Sexes eligible for study: both Accepts healthy volunteers: no Inclusion criteria:  -Subarachnoid haemorrhage confirmed by computed tomography and aneurysm by computed tomography angiography or digital subtraction angiography -Admission to the University Medical Centre Utrecht <12 hours after ictus Exclusion criteria:  - Life expectancy < 10 days - If based on head imaging, it will be unlikely that cerebrospinal fluid can be obtained at day 3 after ictus - Patients with an ongoing infection on admission which is not appropriately treated - Patients on immunosuppressive therapy |
| Study type | Interventional Allocation: randomized Intervention model: parallel assignment Masking: open-label with blinded outcome assessment (PROBE). Outcome assessor is blinded for primary outcome Primary purpose: treatment Phase II |
| Date of first enrolment | October 2018 |
| Target sample size | 26 patients with cerebrospinal fluid samples |
| Recruitment status | Recruiting |
| Primary outcome(s) | Outcome Name: C5a concentration in cerebrospinal fluid Method of measurement: ISO9001 certified multiplex immunoassay based on Luminex technology Timepoint: day 3 after ictus |
| Key secondary outcomes | Outcome Name: Occurrence of adverse events Method of measurement: Listing Timepoint: up to four weeks after ictus  Outcome Name: modified Rankin Score Method of measurement: Telephone interviews will be conducted by a qualified person who is blinded for allocation Timepoint: 13 weeks after ictus (+/- two weeks) |
