# Supplementary material for: CompLement C5 Antibodies for decreasing brain injury after aneurysmal Subarachnoid Haemorrhage (CLASH): study protocol for a randomised controlled phase II clinical trial
Source: Trials. 2020 Nov 25;21:969. doi: 10.1186/s13063-020-04838-6 (PMC7687754; doi:10.1186/s13063-020-04838-6)
Supplement: Supplementary file 2 — Additional file 2. Data management plan. [file 13063_2020_4838_MOESM2_ESM.docx]

Additional File 3

Sampling, processing, storage, and immunoassays

*Blood sampling, processing and storage*
Blood from participants will be collected in lithium heparin tubes for C-reactive protein (CRP) measurements and serum tubes for cytokine levels and functional activity of complement pathways. Upon blood draw, the two serum collection tubes are inverted five times. One of the serum collection tubes is placed on ice with the exception of the serum collection tube drawn on day 1 because logistically this was not possible to arrange in the emergency setting. All samples are immediately transferred to the clinical laboratory of the hospital. The lithium heparin samples will be spun at room temperature 1861g for 10 minutes. The supernatant is used to measure CRP levels. The serum collection tubes will be allowed to stand for 30 minutes after receival at the clinical laboratory and will then be spun at 4 °C 2000g for 10 minutes. The supernatant is collected, aliquoted, and frozen in a –80°C freezer.

*CSF sampling, processing and storage*CSF from participants will be collected in polystyrene collection tubes. The sample tube is immediately placed on ice and transferred to the clinical laboratory where samples are spun at 4 °C 2000g for 10 minutes. The supernatant is collected, aliquoted, and frozen in a –80°C freezer.

*Immunoassays*CRP measurement in serum and CSF will be performed with a turbidimetric immunoassay on a DxAU 5811 automated chemistry analyzer (Beckman-Coulter, Brea, CA, USA). CRP measurements in serum are ISO9001 certified. CRP measurements in CSF are not certified. Functional activity of complement pathways in serum will be measured by enzyme-linked immunosorbent assay (ELISA). Cytokines in serum and CSF will be measured by using an in-house developed and ISO9001 certified multiplex immunoassay (Laboratory of Translational Immunology, University Medical Center Utrecht) based on Luminex technology (xMAP, Luminex Austin TX USA).
